# Gaps in knowledge and use of artemether-lumefantrine among university students in Southwestern Nigeria: A cross-sectional study

**DOI:** 10.1371/journal.pone.0347554

**Published:** 2026-04-20

**Authors:** Edidiong Orok, Oluwaseun Olumoko, Inimuvie Ekada, Amos Oladunni

**Affiliations:** 1 Department of Clinical Pharmacy and Public Health, College of Pharmacy, Afe Babalola University, Ado-Ekiti, Ekiti, Nigeria; 2 Pharmacy Department, Afe Babalola University Multisystem Hospital, Ado-Ekiti, Ekiti, Nigeria; University of Health and Allied Sciences, GHANA

## Abstract

Inappropriate use of antimalarial medications can accelerate the development of antimicrobial resistance (AMR), undermining treatment efficacy and public health goals. Artemether-lumefantrine (A/L) is the first-line treatment for uncomplicated malaria in Nigeria, yet its misuse persists, particularly among young adults. This study assessed knowledge gaps in A/L use among university students in Southwestern Nigeria to identify opportunities for targeted intervention. A cross-sectional online survey was conducted among undergraduate students from three universities in Southwestern Nigeria. Respondents’ knowledge of A/L was categorized as good (≥70%), fair (50–69%), or poor (<50%). Associations between knowledge and demographic or experiential variables were analysed using independent sample t-tests and one-way ANOVA, with significance set at p < 0.05. Data analysis was performed using SPSS version 27. A total of 392 students participated, with a mean age of 19.5 years; 58.4% were female. While 64.5% knew the correct timing for the second dose of A/L, only 22.2% correctly identified appropriate storage methods. About 94.9% preferred swallowing the drug with water after food, but many were unaware of common side effects or appropriate responses. Overall, 44.6% of students demonstrated poor knowledge of A/L use. Knowledge scores differed significantly by level of study (p = 0.001) and previous use of antimalarials (p = 0.04). Substantial knowledge gaps and misconceptions about A/L use exist among university students in Southwestern Nigeria. These findings underscore the urgent need for health education initiatives targeting young adults to promote safe and effective use of antimalarial medications and may inform health education strategies aimed at promoting rational antimalarial use and strengthening malaria control efforts.

## Introduction

In sub-Saharan Africa, malaria remains the foremost cause of morbidity and mortality. It accounts for 40−45% of visits to physicians, 57% of hospitalization days, and 40% of under-5 mortality [1]. According to the WHO World Malaria Report 2022, there were an estimated 619,000 malaria deaths globally in 2021, a slight decrease from 625,000 in 2020, but still significantly higher than the 568,000 deaths in 2019 [2]. Consequently, the increased hospitalisation rate, diagnostic cost and unnecessary medicine use severely affects the economy of these countries [2]. These statistics reveal the extent to which malaria remains a public health emergency and the need for a concerted effort in curbing its effect in health care and the economy at large. The discovery of synthetic antimalarial agent has since been the cornerstone of malaria chemotherapy. This innovation reduced the mortality caused by malaria while increasing the average life expectancy of those in malaria-endemic regions [3]. Ultimately, the success of these antimalarial medications stems from their ability to prevent the resistance from the parasite (*Plasmodium spp*.). Hence, measures need to be put in place to safeguard these drugs to prevent their inefficacy.

Malaria manifests when plasmodium is in its erythrocytic stage, and antimalarial drugs primarily target the plasmodium in this stage. The two most prevalent malaria parasites that case malaria illness (*P. falciparum* and *P. vivax*) differ significantly in their treatment regimens. However, in Nigeria, the illness is caused mostly by *P. falciparum*. The WHO has recommended that artemisinin-based combination therapy (ACT) be used as the first line drug in the treatment of uncomplicated Malaria caused by *P. falciparum* [3]*.* It is also efficacious in malaria caused by non-falciparum parasites [4]*.* This is due to the resistance observed in the older drugs.

The mechanism of action of the ACT is such that it limits the parasites developing resistance. The Artemisinin component is shorter acting, rapidly clearing most of the parasites and often in combination with a longer-acting agent that eliminates the remaining parasites. Some examples of ACTs recommended by WHO include artemether/lumefantrine (A/L), artesunate/amodiaquine, artesunate/mefloquine, dihydroartemisinin/piperaquine, artesunate/pyronaridine, and artesunate/sulfadoxine–pyrimethamine [5]. The major drawback in global malaria eradication is the development of drug-resistant mutations of *P. falciparum*, which result in the inefficacy of drugs in regions where malaria is endemic. Among the medications used for the treatment of malaria, the ACTs are still efficacious in reducing and clearing the parasite counts.

Among the most commonly used antimalarial medications is A/L, which is recommended for the treatment of uncomplicated Plasmodium falciparum malaria [5]. Despite its widespread use, there is growing evidence of therapeutic failure and the development of resistance associated with this medication in West Africa [6,7].

The development of antimalarial drug resistance is not only attributed to genetic mutations of the organisms; self-medication, excessive use or misuse and non-adherence are some of the other causes of this resistance [8,9]. Also, the lack of susceptible testing before prescription plays a critical role in antimalarial resistance [9].

Antimalarial medication misuse and improper use are critical concerns in the fight against malaria, particularly in regions with high disease prevalence like Nigeria [10,11]. Gaps in knowledge and improper use of this medication can also contribute to the development of antimicrobial resistance (AMR) [12].

University students, who represent a significant and educated segment of the population, are expected to have better health literacy. However, evidence has indicated that even among this group, there may be significant knowledge gaps and misconceptions regarding the correct use of antimalarial drugs [13]. Improper usage of antimalarial agents has broader public health consequences because of the potential to accelerate the emergence and spread of resistant malaria strains [14]. Additionally, university students are at a crucial intersection of educational influence and behavioural change. They are likely to be future leaders and influencers in their communities, and their understanding and practices can significantly impact public health. Moreover, university students often serve as a bridge between educated and less educated populations, making them a strategic target for interventions aimed at improving antimalarial use and reducing AMR [15].

Understanding the extent of knowledge gaps and usage patterns of antimalarial medications among university students is crucial. This demographic behaviour and awareness can significantly influence community health practices and policies. The case study of A/L provides a focused lens to examine these issues, given its critical role in current malaria treatment protocols.

A/L is widely recommended by health authorities, including the World Health Organization, for the treatment of uncomplicated malaria due to its high efficacy [5]. This combination therapy helps in delaying the development of resistance, which is crucial in malaria-endemic regions. It is one of the most commonly prescribed antimalarial in Nigeria, making it an ideal candidate for assessing knowledge and usage patterns. Understanding how well this medication is used can provide insights into the effectiveness of current health policies and educational campaigns related to malaria treatment. Therefore, this study aimed to assess the level of knowledge and usage patterns of artemether-lumefantrine among university students in Southwestern Nigeria and to identify factors associated with knowledge gaps.

## Materials and methods

### Study design and duration

A cross-sectional design was conducted online, and a questionnaire was disseminated via the participants’ WhatsApp groups from February 29 to April 3, 2024.

### Study setting and population

The study was carried out among university students in universities in southwestern Nigeria.

### Inclusion and exclusion criteria

Inclusion criteria were based on consent to participate in the study, and those who did not consent were excluded.

### Sample size determination

The total population of students from selected universities were 51000 (Afe Babalola University: 8500; Obafemi Awolowo University: 26000; University of Ibadan: 16500) for 2022/2023 session. A default proportion of 50% (0.5) was adopted for the sample size calculation as per the standard protocol for studies with no previous data. The sample size was calculated to be 382 participants using Raosoft®, an online calculator with a 95% confidence interval and 5% margin of error.

### Sampling technique

Convenience sampling was used to select three universities in southwestern Nigeria based on popularity and population in Nigeria. The universities included Afe Babalola University, Obafemi Awolowo University, and the University of Ibadan. Proportionate allocation was employed to determine the desired number of participants from university (Table 1).

**Table 1:** Proportionate allocation table.

University	Total Population	Proportion	Sample Size
Afe Babalola University	8,500	0.17	65
Obafemi Awolowo University	26,000	0.51	195
University of Ibadan	16500	0.32	122
**Total**	**51000**		**382**

Questionnaires were distributed to departments of the institutions through WhatsApp groups of students, with reminders sent as needed.

### Data collection instrument

Data were collected using a 26-item semi-structured online questionnaire developed following a comprehensive literature review [13–16]. The questionnaire was designed with three main sections:

### Demographics

This section gathered essential demographic information, including age, gender, institution, college/faculty, department, level, ethnicity, and marital status.

### Information on antimalarial prescribed

Participants were asked to specify the brand of antimalarial drugs they used, the number of pills/tablets in a pack, and the dosage form.

### Knowledge assessment on use of antimalarial

This section was made up of 13 knowledge questions that evaluated participants’ previous usage experience, frequency and timing of doses, method of consumption, reactions to the medication, actions taken upon adverse effects, and storage practices. The questions consisted of multiple-choice as well as yes/no formats. Of the 13 questions, 8 were scored knowledge-based items, while the remaining 5 assessed prior use and experiential practices. The maximum possible knowledge score was 8, with each correct answer awarded 1 point and incorrect answers given a score of 0. Based on their scores, students’ knowledge was categorised into three levels: good (≥70%), fair (50–69%), and poor (<50%). The questionnaire is attached as a supplementary file (S1 Appendix).

### Validity and reliability of the instrument

Content and face validity were carefully evaluated to ensure the instrument’s accuracy and comprehensiveness. Internal consistency was assessed through a pre-test involving 10 undergraduate students of Afe Babalola University, Ado-Ekiti, Nigeria, with reliability measured using Cronbach’s Alpha coefficient. The data from these students were excluded from the final analysis. An overall coefficient value of 0.75 was obtained.

### Data collection procedure

The questionnaire was prepared using Google Forms and distributed electronically to student department groups across various universities. The departments were categorised into three groups; Healthcare for departments that offer healthcare based courses such as medicine, pharmacy, public health etc.; Science-based for departments offering science and technology-based courses such as Engineering, Biology, Mathematics, physics, and chemistry, while Arts-based courses comprised of other courses that did not meet the criteria for healthcare and art-based courses such as; law, social/management sciences, and English etc. The recruitment of participants was conducted through direct researcher involvement and university collaboration, where researchers and collaborators disseminated the questionnaires in relevant student WhatsApp groups after obtaining permission and consent. Also, the researchers liaised with student representatives in the various departments, who served as intermediaries to help disseminate the questionnaires. This approach ensured a more comprehensive and representative sample, enhancing validity and reliability.

### Ethical approval and consent to participate

Ethical approval for this study was obtained from the Afe Babalola Multisystem Hospital Health Research Ethics Committee (ABUADHREC) prior to data collection (Approval No.: AMSH/RET/24/001) (S1 File). The ethical approval granted by ABUADHREC was formally accepted by the University of Ibadan and Obafemi Awolowo University; therefore, separate ethical approvals were not required from each participating institution. An online written consent was obtained before study commencement and participant confidentiality and anonymity were strictly maintained throughout and after the study. The study was conducted in accordance with the ethical principles of the Declaration of Helsinki.

### Data analysis

The sociodemographic characteristics obtained were summarised using descriptive statistics, including frequencies and percentages. The differences in knowledge across categorical variables including gender, age, level of study, course of study, previous use of the antimalarial drug, and the time since last use of the antimalarial drug were analysed using the independent sample t-test and one-way analysis of variance (ANOVA). All statistical tests were two-sided, with a significance level set at p < 0.05. Statistical analyses were conducted using Statistical Product and Service Solutions (SPSS), version 27.

## Results

### Demographics and disease profile of the participants

In this study, 392 students from three universities agreed to participate. Among them, 163 (41.60%) were male, and the mean age was 19.53 ± 2.09 years with minimum age of 16 years and maximum age of 27 years. About 17% study arts-based courses while a larger proportion studied science-based courses (138, 35.20%) and healthcare-based courses (188, 48%). The antimalarial medication taking profile of the participants was assessed, and it was reported that only 95.20% had previously taken the antimalarial drug before. One hundred and six students (28.40%) stated that they last took the antimalarial medication ago while 81 (21.70%) took the drug 4–6 months ago. The students also reported that they had taken the drug 1–2 times (177, 47.50%) and 3 times or more (88, 23.60%) during the year. This has been summarized in Table 2. The students also identified Amatem^®^ (147 (39.41%)), Arthemed^®^ (82 (21.98%)) and Coartem^®^ (72 (19.30%)) were the commonest brands of antimalarial medications taken before while Lonart^®^ (66 (17.69%)), Arenax^®^ (4 (1.07%)) and Lumapil^®^ (2 (0.54%)) were least taken by the participants.

**Table 2:** Socio demographics of students.

A.Demographics			Frequency	Percent
**Age (Mean** ±**SD)**			**19.53 ± 2.085 years (Range: 16–27 years)**	
**Gender**	Male		163	41.60
	Female		229	58.40
**Ethnicity**	Yoruba		174	44.40
	Igbo		95	24.20
	Hausa		18	4.60
	Others		105	26.80
Marital status	Single		389	99.20
	Married		3	0.80
**Institution**	ABUAD		105	26.80
	OAU		158	40.30
	UI		129	32.90
**Courses of study**	Healthcare (e.g., Pharmacy, Medicine, etc.)		188	48
	Arts-based (e.g., Law, English, etc.)		66	16.80
	Science-based (Biology, Physics etc.)		138	35.20
**Level of study**	100-300		211	53.80
	400-600		181	46.20
**B. Antimalarial medication taking profile**				
**Item**		**Frequency**	**Percent**	
**Have you used A/L before? (N = 392)**	Yes	373	95.20	
	No	19	4.80	
**How long ago have you used the antimalarial drug?** **(N = 373)**	<1 month ago	106	28.40	
	1-3 months ago	107	28.70	
	4-6 months ago	81	21.70	
	> 6months ago	79	21.20	
**Dosage form prescribed** **(N = 373)**	Capsules	13	3.50	
	Tablets	360	96.50	
**How often have you used the drug this year** **(N = 373)**	None	108	28.90	
	1-2 times	177	47.50	
	3 times and more	88	23.60	

A/L: Arthemether Lumefantrine; SD: Standard Deviation; ABUAD: Afe Babalola University; OAU: Obafemi Awolowo University; UI: University of Ibadan; Level of study represents participants’ academic year in the university (100 = 1^st^ year; 600 level = 6^th^ year and so on).

### Students’ knowledge of usage of artemether-lumefantrine

Table 3 summarises the students’ knowledge of usage of artemether-lumefantrine in this study. When asked the best time to start taking the antimalarial medicine the responses obtained included after 2 hours (68/392, 17.30%), after food (8/392, 2%), immediately (227/392, 57.90%).

**Table 3:** Students’ knowledge of correct usage of artemether-lumefantrine.

S/N	Statement (N = 392)			Responses (%)	
				Correct	Incorrect
1	When should you start taking the medicine after the pharmacist gives it to you	After 2 hours	68 (17.30%)	14 (3.50)	378 (96.50)
		After food (**correct answer)**	8 (2%)		
		Immediately	227 (57.90%)		
		As instructed by the pharmacist **(correct answer)**	6 (1.50%)		
		Anytime	20 (5.10%)		
		When symptoms worsen	3 (0.80%)		
		The next day	60 (15.30%)		
2	When should you take the second dose of the medication?	24 hours after first dose	16 (4.10%)	253 (64.50)	139 (35.50)
		12 hours after first dose	110 (28.10%)		
		8 hours after first dose **(correct answer)**	253 (64.50%)		
		Immediately after first dose	9 (2.30%)		
		When symptoms worsen	4 (1.10%)		
3	How long does it take to complete a course of the medication?	2 days	13 (3.30%)	273 (69.60)	119 (30.40)
		3 days (**correct answer)**	273 (69.60%)		
		4 days	31 (7.90%)		
		5 days	47 (12%)		
		I never complete the dose	26 (6.60%)		
4	How should you take the medicine?	Put in food	6 (1.50%)	372 (94.90)	20 (5.10)
		Swallow with water after food (**Correct answer**)	372 (94.90%)		
		Swallow with drinks	7 (1.80%)		
		Swallow with milk	7 (1.80%)		
5	What possible side effects could you feel after taking this drug? *	Dizziness	56 (14.30%)	125 (31.90)	267 (68.10)
		Nausea	3 (0.80%)		
		Vomiting/Diarrhoea	50 (12.80%)		
		Sleep disturbances	12 (3.10%)		
		Body weakness	4 (1.00%)		
		**(All answers are correct)**			
6	What should you do when experiencing any side effects?	Nothing	299 (76.30%)	37 (9.40)	355 (90.60)
		Get adequate rest/sleep	7 (1.80%)		
		Report to prescriber (**Correct answer)**	37 (9.40%)		
		Seek further treatment	49 (12.50%)		
7	What do you do if you vomit immediately after taking the medication	I don’t know	35 (9%)	69 (17.60)	323 (82.40)
		Take the next dose **(Correct answer)**	69 (17.60%)		
		Meet the prescriber	3 (0.80%)		
		Stop taking the medicine	100 (25.50%)		
		Take with next dose	185 (47.2%)		
8	How do you store this medication while at home?	In the cupboard **(Correct answer)**	254 (64.8%)	254 (64.80)	138 (35.20)
		In my bag	46 (11.7%)		
		In an open space	90 (23.0%)		
		Under the bed	2 (0.5%)		
9	Number of tablets to be taken			286 (73.00)	106 (27.00)
	**Poor Knowledge (<50% total knowledge score)**			175 (44.60)	
	**Fair Knowledge (50%−69% total knowledge score)**			200 (51.00)	
	**Good Knowledge (≥ 70% total knowledge score)**			17 (4.30)	

Also, majority of the students (253/392, 64.50%) stated that the second dose of the medication should be taken 8 hours after the first dose while 9/392 (2.30%) reported that it should be taken immediately after the first dose. Thirty-one students (7.90%) reported that it would take then 4 days to complete the medication while 13/392 (3.30%) stated that it takes 2 days. More than 6.50% (26/392) reported that they would never complete the dose and that they would stop immediately after feeling better. When asked about how best they should take their medication, 372/392 (94.90%) stated that they would swallow with water after food and 7/392 (1.8%) reported that they would swallow with drinks and milk. The students identified vomiting/diarrhoea (50/392 (12.80%)), sleep disturbances (12/392 (3.10%)), body weakness (4/392 (1.00%)) as the possible side effects associated with the medication. They also reported that they would get adequate rest/sleep (7/392 (1.80%)), report to prescriber (37/392 (9.40%)), stop taking medicine (100/392 (25.50%)) and taking the next dose (185/392 (47.20%)) as steps they would take when faced with side effects of the medication.

One hundred students (25.50%) reported that they would stop taking the medication if they are to vomit immediately after taking the antimalarial drug while 47.20% (185/392) reported that they would take two doses during time for the next dose. A total of three hundred and two students (77%) did not know the proper way of storing the antimalarial medication. About 65% (254/392) stated that they would store the drug in the cupboard while 90/392 (23%) opted to storing the medicine in an open space. Overall, 175/392 students (44.60%) of the students showed poor knowledge of usage of the antimalarial drugs prescribed to them.

Table 4 shows statistical differences in knowledge scores based on demographics, and antimalarial medication taking profile of students. There was a statistically significant difference in knowledge scores based on student levels (p = 0.001) and previous usage of antimalarial drug (p = 0.04) by the students. Also, female students (p = 0.07), students of higher age (p = 0.27) and healthcare students (p = 0.28) had better knowledge than male students, students of lower age and students in other disciplines respectively but this was not statistically significant.

**Table 4:** Differences in knowledge scores based on demographics, and antimalarial medication taking profile of students.

Variables		Frequency (%)	Mean score ± SD	p-value
Gender	Male	163 (41.60)	3.74 ± 1.225	0.070^a^
	Female	229 (58.40)	3.97 ± 1.188	
Age	16-19 years	203 (51.80)	3.78 ±1.233	0.270^b^
	20-23 years	171 (43.60)	3.98 ± 1.191	
	Above 23 years	18 (4.60)	3.89 ± 1.023	
Level of study	100-300	211 (70.80)	3.69 ± 1.229	**<0.001** ^**a**^ *
	400-600	181 (29.20)	**4.08 ± 1.149**	
Courses of study	Science-based	138 (35.20)	3.77 ± 1.142	0.280^b^
	Arts-based	66 (16.80)	3.80 ± 1.417	
	Healthcare	188 (48)	3.97 ± 1.172	
Have you used this antimalarial before?	Yes	373 (95.20)	**3.90 ± 1.202**	**0.040** ^**a**^ *
	No	19 (4.80)	3.32 ± 1.204	
How long ago have you used this antimalarial drug?	<1 month	106 (27)	3.93 ± 1.181	0.090^**b**^
	1-3 months	107 (27.30)	4.06 ± 1.243	
	4-6 months	81 (20.70)	3.75 ± 1.230	
	>6 months	79 (20.20)	3.79 ± 1.137	
	None	19 (4.80)	3.32 ± 1.204	

SD: Standard deviation; ^a^: Independent Sample T-test; ^b^: One Way Analysis of Variance; *: Statistically significant value, p ≤ 0.05.

## Discussion

Malaria remains a significant public health challenge in tropical and subtropical regions, particularly in sub-Saharan Africa, where it is a leading cause of morbidity and mortality [17,18]. Although the use of antimalarial medications was initially effective in malaria control, the emergence of chloroquine resistance prompted a global shift toward ACTs [19,20]. However, emerging resistance to ACTs is now a concern, especially in parts of Asia and Africa [17,21]. These efforts to contain resistance could be undermined if ACTs, including A/L, are misused or inappropriately used.

This study assessed university students’ knowledge and practices concerning A/L use, with the goal of identifying gaps that could inform public health interventions. The findings demonstrate a high prevalence of A/L use among students, which may reflect both a high malaria burden and a tendency toward self-treatment. Similar results were reported in a study in Northeast Ethiopia, which found a 30% prevalence of ACT use [22], and in Southwestern Nigeria, where ACTs were the second most self-prescribed medications among university students [23]. These findings confirm the endemic nature of malaria and the widespread use of antimalarial medications among young adults. Varying levels of exposure of the students were also noticed as 28.40% of students reported using A/L less than a month ago. In addition, almost half of the students (37.50%) had taken A/L once or twice and 23.60%, three times or more within the year which was similar to the usage of ACT in another student conducted in Nigeria that revealed 83% of ACT usage by university students [24]. This result raises concerns on the possibility of relapse or inappropriate usage of ACT especially A/L among university students.

Notably, 28.4% of students reported using A/L within the last month, and over 60% had taken the medication at least once in the past year. While frequent usage might suggest experience with malaria, it also raises concerns about potential misuse or overuse, especially in the absence of diagnostic confirmation. These patterns of recurrent use point toward a fundamental knowledge gap.

Alarmingly, only 4.3% of respondents demonstrated good knowledge of A/L usage. Despite widespread use, significant misconceptions persist, highlighting the need for improved public education. For instance, a study in Ghana found that 73% of respondents believed ACTs had no side effects [25], whereas Afaya et al. [16] reported that 79% had good knowledge of ACTs. In the present study, students with prior experience using A/L had significantly higher knowledge scores, suggesting that first-hand experience may improve understanding, although it is not a substitute for formal education. The study also found that a large proportion of higher-level students (90.6%) were unaware of the appropriate actions to take when experiencing side effects. Similarly, 82.4% of participants did not know the correct procedure to follow if vomiting occurred shortly after taking A/L. According to treatment guidelines, a repeat dose is recommended if emesis occurs within one to two hours of administration [26]. These findings suggest an urgent need to integrate basic treatment literacy into student health education.

A statistically significant difference was found in knowledge scores between students of different academic levels, with higher-level students performing better. This aligns with previous studies [22,24], possibly due to increased exposure to health-related coursework and experience. Many students in this study were enrolled in health-related disciplines, a trend consistent with other Nigerian and African research [22–24]. Being in a healthcare program likely contributed to better understanding of ACTs.

### Limitations of study

This study, while providing valuable insights into the knowledge and practices related to antimalarial medication use among university students, has several limitations. Firstly, the use of an online questionnaire distributed via WhatsApp groups may have introduced sampling bias, potentially excluding students without access to smartphones or the internet. This limitation could impact the generalizability of the findings to the broader student population. Also, the reliance on self-reported data poses another limitation. Participants’ responses may be influenced by recall bias or inaccuracies in their understanding of their own medication practices and knowledge.

The cross-sectional design of the study provides only a snapshot of students’ knowledge and behaviours at a single point in time. This design does not account for how knowledge and practices might evolve over time. Longitudinal studies could offer deeper insights into these changes and their impacts on antimalarial medication use.

Furthermore, the questionnaire may not have captured the full depth of participants’ knowledge about antimalarial medications. A more comprehensive assessment, including qualitative methods such as interviews or focus groups, could yield more detailed information on participants’ understanding and practices. The study’s findings are also limited in their generalizability. Conducted among university students in southwestern Nigeria, the results may not be applicable to other regions or populations.

Additionally, while the study considered prior antimalarial usage, it did not account for variations in the type or duration of previous treatments. Such factors could influence current knowledge and practices, suggesting a need for more detailed data on this aspect. The study did not assess laboratory-confirmed malaria diagnosis, Plasmodium species, or prescriber source, as the focus was on knowledge and reported use patterns. As with all self-administered online surveys, the possibility of participants consulting external sources while completing the questionnaire cannot be entirely excluded.

## Conclusion

This study identified critical knowledge gaps and misconceptions regarding the use of artemether-lumefantrine among university students in Southwestern Nigeria. Despite widespread use, awareness of proper dosage, side effects, and storage practices remains low. These knowledge deficiencies may predispose to inappropriate drug use, which has been associated in the literature with increased risk of treatment failure and resistance emergence. Targeted health education and awareness campaigns within university settings are urgently needed to promote responsible antimalarial use and safeguard the efficacy of ACTs. Future studies should explore the effectiveness of educational interventions in improving knowledge and behaviour around antimalarial drug use.

## Supporting information

S1 Appendix Data collection instrument. (PDF)

S1 File Ethical approval for the study. (PDF)
