# Supplementary material for: Gaps in knowledge and use of artemether-lumefantrine among university students in Southwestern Nigeria: A cross-sectional study
Source: PLoS One. 2026 Apr 20;21(4):e0347554. doi: 10.1371/journal.pone.0347554 (PMC13094982; doi:10.1371/journal.pone.0347554)
Supplement: S1 Appendix — (PDF) [file pone.0347554.s001.pdf]

Assessing the knowledge of correct usage of Antimalarial Drugs among University Students in Southwestern Nigeria: A Case Study using artemether-lumefantrine

Artemether-Lumefantrine (AL) is a combination antimalarial drug widely used for the treatment of uncomplicated malaria caused by Plasmodium falciparum.

Misuse or inadequate understanding of antimalarial drugs can lead to treatment failure, the development of drug resistance, and potential harm to individuals. This research is important as it will ensure that students, as potential users of antimalarial medications, possess accurate knowledge about the correct usage of Artemether-Lumefantrine.
The study will involve collecting data on students' awareness of Artemether-Lumefantrine, understanding of its dosing regimen, knowledge about potential side effects, and adherence to prescribed treatment duration. Findings from this research will contribute to the existing literature on antimalarial drug knowledge and inform educational interventions to improve the proper usage of these medications among the student population.
We kindly request your participation in this study by completing the form, which will take less than 10 minutes of your time. Your participation is crucial in providing valuable insights into the treatment of uncomplicated malaria. **Thank you.**

* Indicates required question

1. Do you consent to participate in this study? *

Mark only one oval.

- Yes
- No

Demographics

This section will document the demographics of the students

2. Age *

____

3. Gender *

Mark only one oval.

- Male
- Female

4. Name of Institution *

Mark only one oval.

- ⬭ Afe Babalola University, Ado-Ekiti
- ⬭ Obafemi Awolowo University, Ile-Ife
- ⬭ University of Ibadan

5. College/Faculty *

Mark only one oval.

- ⬭ Pharmacy
- ⬭ Engineering
- ⬭ Sciences
- ⬭ Law
- ⬭ SMS
- ⬭ MHS
- ⬭ Other: ____________________

6. Department

____

7. Level *

Mark only one oval.

- ⬭ 100
- ⬭ 200
- ⬭ 300
- ⬭ 400
- ⬭ 500
- ⬭ 600

8. Ethnicity *

Mark only one oval.

- Yoruba
- Igbo
- Hausa
- Other: ____________________

9. Marital Status *

Mark only one oval.

- Single
- Married

INFORMATION ON ANTIMALARIAL PRESCRIBED

10. Brand of the antimalarial *

Mark only one oval.

- Arthemed
- Amatem
- Coartem
- Lonart
- Arenax
- Other: ____________________

11. Number of pills/tablets in a pack *

Mark only one oval.

- 6
- 12
- 18
- 24

12. Dosage form prescribed *

Mark only one oval.

- Tablets
- Suspension
- Other: ______________________

Knowledge Assessment on use of Antimalarial

13. Have you used this medicine for malaria before? *

Mark only one oval.

- Yes
- No

14. How long ago have you used the antimalarial? *

Mark only one oval.

- <1 month ago
- 1- 3 months ago
- 4-6 months ago
- >6 months ago

15. How often have you used these drugs during this year? *

Mark only one oval.

- 1-2 times
- 3-4 times
- 4 times
- None

16. How many tablets do you take per given time? *

Mark only one oval.

- 1
- 2
- 3
- 4

17. When do you start taking your medicine after the pharmacist gives it to you? *

Mark only one oval.

- Immediately
- After 2hours
- The next day
- Only when my symptoms worsen
- Anytime
- Other: ______________________

18. When do you take your second dose after leaving the pharmacy? *

Mark only one oval.

- After 8hours
- After 12hours
- When I feel my symptoms are getting worse
- Immediately after the first dose
- 24 hours after the first dose
- Other: ______________________

19. How long do you take to complete taking all the medicines given to you? *

Mark only one oval.

- 2 days
- 3 days
- 4 days
- 5 days
- I never complete the dose
- Other: ______________________

20. How do you take this medicine? *

Mark only one oval.

- Swallow with water
- Swallow with soft drinks
- Swallow with milk
- Crush and dissolve in water
- Put it in food

21. What do you take with this medicine? *

Mark only one oval.

- Water
- Alcohol
- Fruit Juice
- Soft drinks e.g Coca-Cola, Fanta
- Energy drinks e.g Predator
- Other: ____________________

22. How do you feel after taking this drug? *

Mark only one oval.

- Dizziness
- Vomiting
- Disturbed sleep
- I have no problem with this drug
- Other: ____________________

23. What do you do when you experience any of the above while taking this drug? *

Mark only one oval.

- I do not worry as they disappear shortly
- I seek further treatment
- Report to the prescriber
- Other: ____________________

24. What do you do when you vomit immediately (within 2 hours) after taking this medicine? *

Mark only one oval.

- ( ) I stop taking the medicine
- ( ) I wait for the next dose
- ( ) I take a new dose
- ( ) Other: ____________________

25. How do you keep your medicines while at home? *

Mark only one oval.

- ( ) In my hand bag
- ( ) Open space
- ( ) In its pack inside the cupboard
- ( ) Under my bed

This content is neither created nor endorsed by Google.

Google Forms
