# Supplementary material for: Gaps in knowledge and use of artemether-lumefantrine among university students in Southwestern Nigeria: A cross-sectional study
Source: PLoS One. 2026 Apr 20;21(4):e0347554. doi: 10.1371/journal.pone.0347554 (PMC13094982; doi:10.1371/journal.pone.0347554)
Supplement: S1 File — (PDF) [file pone.0347554.s002.pdf]

AFE BABALOLA UNIVERSITY MULTI-SYSTEM HOSPITAL
Km 8.5, Afe Babalola Way, Ado Ekiti, Ekiti State, Nigeria
Website: www.amsh.abuad.edu.ng
Tel: +234 907 887 6353, +234 803 718 0715, +234 909 321 4097, +234 808 813 7062, +234 806 618 6037 Email: amsh@abuad.edu.ng

Ref. No: AMSH/RET/24/001 12th February 2024

Oluwaseun Goodness Olumoko,
ABUAD Pharmacy,
Afe Babalola Multisystem Hospital,
Ado-Ekiti, Ekiti State.

Sir/Ma,

RESEARCH ETHICAL APPROVAL

I am writing to inform you that your request for research ethical approval has been **approved**.

You may proceed with your study titled *"Assessing The Knowledge Of Afe Babalola University Students On The Correct Usage of Antimalarial Drugs With Artemether-Lumefantrine As a Case Study."*

We wish you success in your study.

Dr. Fadlulai Abdu-Raheem, *MD, Postgr. MD, FMCPaed*

Chairman, Research and Ethics Committee
